# Glass-Ceramic Foams from ‘Weak Alkali Activation’ and Gel-Casting of Waste Glass/Fly Ash Mixtures

**DOI:** 10.3390/ma12040588

**Published:** 2019-02-15

**Authors:** Acacio Rincón Romero, Nicoletta Toniolo, Aldo R. Boccaccini, Enrico Bernardo

**Affiliations:** 1 Department of Industrial Engineering, University of Padova, Via Marzolo 9, Padova 35131, Italy; acacio.rinconromero@unipd.it; 2 Institute of Biomaterials, Department of Materials Science and Engineering, University of Erlangen-Nuremberg, Cauerstraße 6, 91058 Erlangen, Germany; nicoletta.t.toniolo@fau.de (N.T.); aldo.boccaccini@ww.uni-erlangen.de (A.R.B.)

**Keywords:** alkali activation, inorganic gel casting, glass–ceramic foams, waste glass, fly ash

## Abstract

A ‘weak alkali activation’ was applied to aqueous suspensions based on soda lime glass and coal fly ash. Unlike in actual geopolymers, an extensive formation of zeolite-like gels was not expected, due to the low molarity of the alkali activator (NaOH) used. In any case, the suspension underwent gelation and presented a marked pseudoplastic behavior. A significant foaming could be achieved by air incorporation, in turn resulting from intensive mechanical stirring (with the help of a surfactant), before complete hardening. Dried foams were later subjected to heat treatment at 700–900 °C. The interactions between glass and fly ash, upon firing, determined the formation of new crystal phases, particularly nepheline (sodium alumino–silicate), with remarkable crushing strength (~6 MPa, with a porosity of about 70%). The fired materials, finally, demonstrated a successful stabilization of pollutants from fly ash and a low thermal conductivity that could be exploited for building applications.

## 1. Introduction

Coal fly ash is a fundamental waste produced by power stations; it consists of a fine particulate material with fluctuating chemical and phase compositions, depending on the original coal and burning conditions, configuring a significant environmental issue. In fact, despite the exploitation of renewable energy resources, the amounts of waste generated worldwide are increasing (the currently produced amount, of about 900 million tons, is expected to increase up to 2 billion tons in 2020) [1]. Coal fly ash is mainly landfilled, producing significant dangers, such as the potential leaching of heavy metals or polycyclic aromatic hydrocarbons [2,3,4]. As a consequence, the use of coal fly ash in new valuable materials is a critical issue for a sustainable society.

Significant amounts of fly ash are used in the building industry, due to their pozzolanic properties: mixed with cement, they are well known to improve concrete durability [3,5,6]. Fly ash valorization has also been realized by the production of dense glass–ceramic materials, to be used as an alternative to natural stones or traditional ceramic tiles [7,8].

Compared to dense glass–ceramics, lightweight glass–ceramic foams, to be used for thermal and acoustic insulation, may represent a more valuable product. Waste glass/fly ash mixtures have been variously foamed, by viscous flow sintering of glass, determining a pyroplastic mass (in turn incorporating fly ash), with concurrent gas evolution, by the addition of different foaming agents, such as SiC [9]. The amount and nature of waste glass are significant in reducing the processing temperature [10,11]. As an example, carbonates, in the form of dolomite (CaMg(CO_3_)_2_), or sludge from a marble cutting–polishing plant (containing mainly calcite, CaCO_3_) may lead to ceramic foams with low apparent density (0.36–0.41 g/cm^3^) and relatively high compressive strength values (2.4–2.8 MPa). However, the highest amount that could be incorporated for the foam production does not exceed 20 wt% [3]. Coal fly ash may be increased up to 40 wt%, in foams generated by means CaCO_3_ decomposition, but 30 wt% borax must be considered as extra fluxing agent [11]. Some attempts to produce lightweight aggregates have been made even avoiding the use of any foaming agents, e.g., by mixing the fly ash (75 wt%) and waste window panes (25 wt%) exploiting the bloating of the fly ash at high temperature [12].

According to the fact that fly ash is rich in amorphous alumina and silica, another promising application concerns the formulation of waste-derived geopolymers [13]. Geopolymer materials are a class of inorganic polymers synthesized through the reaction of a solid alumino–silicate precursor with a highly concentrated alkali solution. When immersed in strongly alkaline solutions, the alumino–silicate-reactive materials are almost entirely dissolved, leading to the formation of hydrated alumino–silicate oligomers, including [SiO_4_] and [AlO_4_] tetrahedral units. The following polymerization of the oligomers creates a highly stable three-dimensional network structure where the [SiO_4_] and [AlO_4_] tetrahedra are linked together by sharing oxygen atoms. This structure (‘zeolite-like gel’) resembles that of zeolites, but it mostly reveals an amorphous nature [14].

The use of fly ash in the geopolymer formulation, alone or mixed with other natural sources or industrial wastes, has been widely studied [15,16,17], with the perspective of obtaining inorganic binders with low CO_2_ emissions (compared to ordinary Portland cement) and reduced use of natural raw materials [18,19]. These advantages, however, are somewhat counterbalanced by the costs of the standard reagents used as alkali activators in fly ash geopolymer-based materials, such as sodium silicate (also known as water glass) and sodium or potassium hydroxide [20,21]. In fact, it should be noted that significant energy demand and CO_2_ emissions are associated with sodium silicate production, where temperatures around 1300 °C are required, in order to melt sodium carbonate and silica mixtures [22]. Some efforts have recently been made to substitute the conventional activators with low cost and more environmentally friendly alternatives, such as industrial residues of discarded cleaning solutions for aluminum molds [23] and, above all, waste glass [24]. 

In recent investigations, we proposed the synthesis of dense fly ash [25] or red mud [26] based geopolymers using waste glass from municipal waste collection, replacing water glass as main silica source in the geopolymeric formulation. It was established that, despite the alternative formulation, stable and chemically resistant geopolymeric gels could be obtained by mixing fly ash and waste glass, in an appropriate ratio, and using NaOH in relatively low molarities (NaOH 8M) [25]. The present investigation was conceived as a further extension of the approach, in the development of inorganic gels with an even weaker alkali activation of waste glass/fly ash aqueous suspensions. The suspensions were subjected to extensive foaming (by mechanical stirring) at the early stage of gelation, leading to highly porous bodies later stabilized by means of a low temperature firing treatment.

## 2. Materials and Methods

The initial raw materials used in this study were low calcium fly ash (FA) class F (ASTM C 618) [27], with a mean particle size of 20 µm, supplied by Steag Power Minerals (Dinslaken, Germany), and soda–lime glass waste (SLG), produced by SASIL S.r.l. (Brusnengo, Biella, Italy) as fine powders with a particle size under 30 µm. In particular, we considered the finest fraction produced during the purification process of glass cullet carried out in the company, with limited industrial application. Table 1 summarizes the chemical composition of the two basic raw materials determined by means of X-ray fluorescence [25].

FA/SLG were prepared by progressive addition of powders into an NaOH aqueous solution, for a liquid/solid of 0.45. According to a methodology already presented in previous studies [25,26], we varied the theoretical molar ratio between SiO_2_ and Al_2_O_3_ in the final product by changing the FA and SLG proportions, being 76/24, 64/36, 54/46, corresponding to a SiO_2_/Al_2_O_3_ theoretical molar ratio of 5, 6, and 7, respectively. However, in this case, a lower concentration (3M) of NaOH was considered. As in previous studies, the mixtures were kept under low-speed mechanical stirring (500 rpm) for 4 h, to ensure a good dissolution of the starting materials and the proper dispersion of the remaining undissolved particles in the slurries.

**Figure 1:**
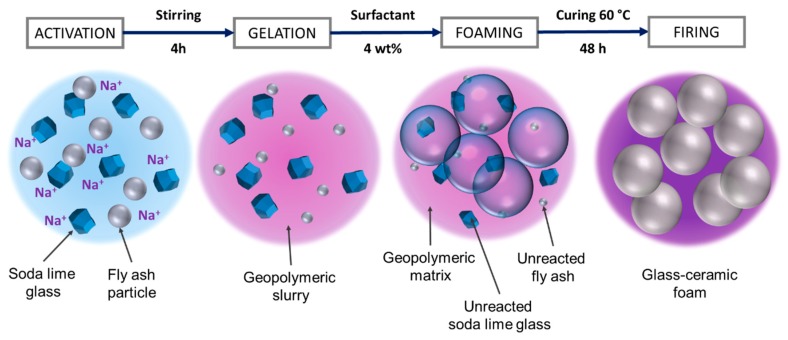
Processing scheme for the production of glass–ceramic foams.

The suspensions were foamed by the addition of 4 wt% of an aqueous solution of sodium lauryl sulphate (SLS) (CH_3_(CH_2_)_11_OSO_3_Na, supplied by Carlo Erba, Cornaredo, Milan, Italy), previously prepared with a SLS/water = 1/10 and then by application of vigorous mechanical stirring (2000 rpm) for 10 min. The prepared wet foams were poured into cylindrical molds (6 cm diameter) and kept at 60 °C for 48 h in sealed conditions. Dried samples were finally demolded and subjected to thermal treatment at 800, 900, and 1000 °C for 1 h (10 °C/min heating rate). The overall manufacturing process is represented by Figure 1.

X-ray diffraction analyses (XRD) were carried out on powdered samples (Bruker D8 Advance, Karlsruhe, Germany) using CuKα radiation (0.15418 mm), 40 kV-40mA, and 2Ɵ angles between 10–70°. A step size of 0.02° and 2 s counting time was set for the analysis of the starting materials and of the hardened foams. A step size of 0.02° and a 0.5 s counting time was adopted for the fired samples. The phase identification was performed using the Match!^®^ software suite (Crystal Impact GbR, Bonn, Germany), supported by data from PDF-2 database (ICDD- International Centre for Diffraction Data, Newtown Square, PA, USA).

Samples form larger specimens were cut to about 10 × 10 × 10 mm. The bulk density was evaluated considering the mass measured with an analytical balance and the volume carefully measured with a digital caliper. The apparent and true densities were determined using a helium pycnometer (Micromeritics AccuPyc 1330, Norcross, GA, USA), working on foamed samples or on fine powders from crushed samples, respectively. The total, open, and closed porosity were computed using the three density values. Compression tests were done using an Instron 1121 UTS (Instron, Danvers, MA, USA) machine, with a crosshead speed of 0.5 mm/min, with each data point corresponding to 9–10 samples.

The European Standard for the compliance test for leaching of granular waste materials and sludge (EN 12457-2) was followed to evaluate the release of heavy metals in the initial raw materials and in selected fired samples. Fragments below 4 mm were placed in distilled water with a pH value of ~7 to a liquid/solid ratio of 10, softly stirred at 25 °C for 24 h. The resulting eluates were filtered through a 0.6 µm filter and analyzed using inductively coupled plasma (ICP; SPECTRO Analytical Instruments GmbH, Kleve, Germany).

The semi-industrial production of lightweight panels was developed for selected samples. The alkali activation process was conducted in the same way. However, a relatively significant amount of activated suspension was prepared for each batch (around 5 kg), and the process was carried out with technical grade reagents. The hardened foamed panels were obtained after casting the wet foams in bigger molds (15 cm × 20 cm). Finally, hardened foamed gels were fired at 800 °C for 1 h, using a pilot scale tunnel furnace (Nanetti ER-15S) at SASIL S. p. a. (Brusnengo, Biella, Italy).

Thermal conductivity tests on the lightweight panels were performed using a Fox 50 Heat Flow Meter by TA Instruments (New Castle, DE, USA) operating at 25 °C. The measure was conducted on cylindrical samples (50 mm diameter and 10 mm thickness) cut from the panels; three replicated tests were performed on samples taken from different panels.

## 3. Results and Discussion

**Figure 2:**
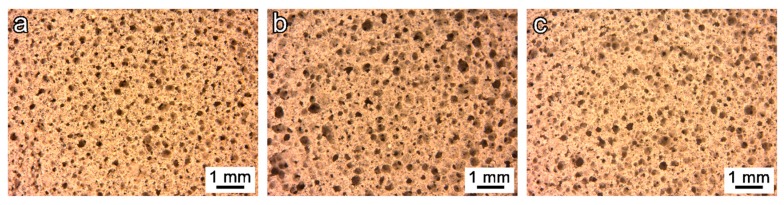
Microstructural details of the soda–lime glass waste/fly ash (SLG/FA foams in the hardened state; (**a**) 5S; (**b**) 6S, and (**c**) 7S.

Figure 2 shows the microstructure for the geopolymer foam after 24 h of curing for the mixtures 5S, 6S, and 7S (Figure 2a–c, respectively). The hardened foams reveal a high microstructural uniformity, presenting pores in a range from 10 to 30 μm diameter. No significant changes in the pore distribution between samples with different compositions can be detected, even if the viscosity of the initial slurries was different. High amounts of glass were expected to determine a slight viscosity increase [28], but the spherical morphology of the FA ensured good workability in all conditions; the observed great homogeneity presented can be explained thanks to the rapid setting of the wet foams, preventing any coalescence effect [29].

**Figure 3:**
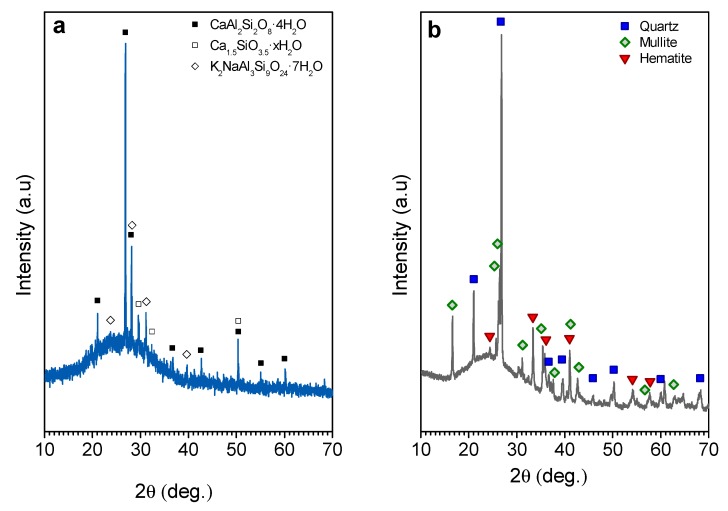
X-ray diffraction patterns of the initial materials (**a**) waste soda lime glass and (**b**) fly ash.

The X-ray diffraction patterns of the starting materials are illustrated in Figure 3; a different intensity scale was selected for each material in order to highlight the crystalline phases present (the intensity of the strongest peak in Figure 3b is ~20 times higher than the intensity of the strongest peak in Figure 3a). SLG (Figure 3a) presents the typical ‘amorphous halo’ of silicate glasses centered approximately at 2θ = 24–26°, along with weak peaks, attributed to hydrated phases, such as calcium aluminum silica hydrate (gismondine CaAl_2_Si_2_O_8_·4H_2_O, PDF#020-0452), calcium silica hydrate (Ca_1.5_SiO_3.5_·xH_2_O, PDF#033-0306), and sodium aluminum silica hydrate (K_2_NaAl_3_Si_9_O2_4_·7H_2_O, PDF#022-0773). These hydrated phases had formed probably according to surface reaction of fine glass particles with environmental humidity during storage.

The original fly ash (Figure 3b) contained quartz (SiO_2_, PDF#083-0539) and mullite (Al_4_SiO_8_, PDF#079-1275) as the main crystal phases; moreover, minor traces of hematite (Fe_2_O_3_, PDF#033-0664) were also detected. In the pattern, it is still possible to detect an amorphous halo at around 24–26°.

**Figure 4:**
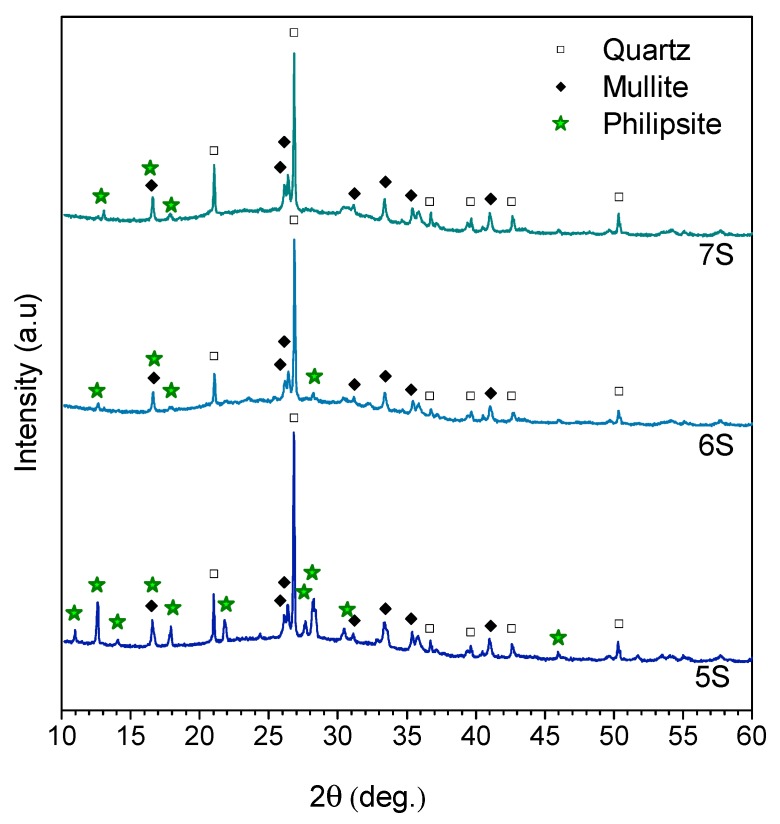
X-rays diffraction patterns of the hardened geopolymeric foams.

The X-ray diffraction patterns of the hardened foams represented in Figure 4 show that quartz and mullite from the initial fly ash remained practically unaltered. The intensity of these peaks simply decreased with a higher amount of glass in the initial formulation; as a result of this ‘dilution’ effect, hematite (Fe_2_O_3_, PDF#033-0664) is no longer visible.

The center of the amorphous halo moved from 2θ~24°, in the original raw materials, to 2θ~30°, after alkali activation. This shift is consistent with the literature on geopolymers, concerning the development of an alkaline alumino–silicate hydrate (N–A–S–H) gel as a reaction product [30,31,32]. 

A second change, with alkali activation, concerns the presence of philipsite (Na_4_KAl_5_Si_11_O_32_(H_2_O)_10_, PDF#073-1419), as newly formed crystal phase, for all compositions. This phase, a sodium potassium aluminum silicate hydrate with a zeolite-type structure, has already been detected in geopolymeric gels [33,34]. Although quite rough, both shift of amorphous halo and formation of philipsite could be considered as a proof of the reaction between starting materials and development of a semi-crystalline ‘geopolymer-like’ gel.

**Figure 5:**
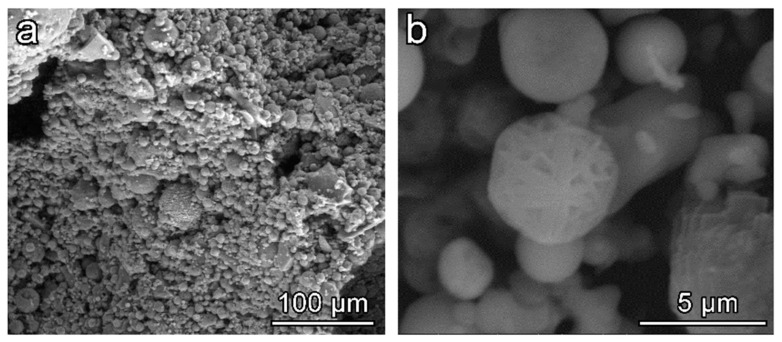
High magnification morphology of 5S hardened state foam: (**a**) Low magnification; (**b**) high magnification.

Microstructural details of the cell struts from hardened sample 5S were studied using SEM (Figure 5). The low magnification image (Figure 5a) reveals the spherical shape of the fly ash mixed with irregular shape particles of SLG. As could be noticed, the low activator molarity did not allow a significant dissolution of the initial raw materials. However, the reaction products detected in the X-ray analysis effectively promoted the binding of adjacent particles in the hardened samples. At higher magnification, zeolite crystals were well visible on the surface of the unreacted particles (Figure 5b). The particular morphology of deposits on spherical fly ash particles is typically associated with the formation of zeolite compounds [35,36].

The hardened foams kept their structural integrity after drying. However, after immersion in boiling water, the water became turbid, and pH rapidly approached 13. This fact revealed the poor chemical stability of the hardened samples when activated at low molarity and justified the application of a firing treatment for further stabilization.

**Figure 6:**
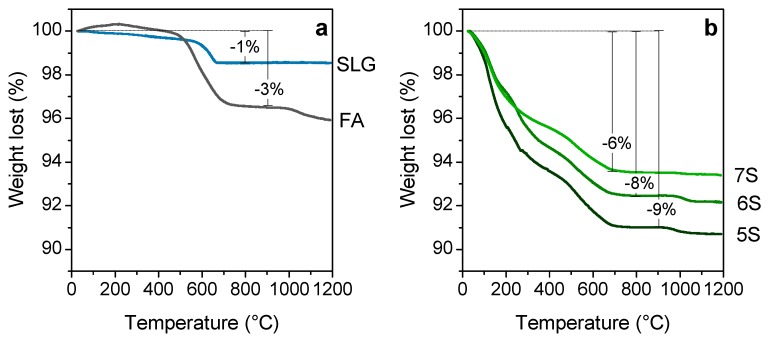
Differential thermal analysis plots; (**a**) initial soda lime glass cullet and fly ash; (**b**) hardened foams.

The weight losses of the initial SLG waste and FA are presented in Figure 6a. The waste glass presents a negligible loss of weight around 1 wt% at 700 °C, which is attributed to the burn-out of the plastic impurities inside the waste glass fraction. The weight losses of the FA are approximately 4 wt% in the temperature range studied (20–1200 °C). Losses between 200 and 800 °C are associated with the combustion of carbon present in the FA, and marginal loss of near 1 wt% at 1000 °C is associated with sulphate decomposition [37].

The TGA results of the hardened samples shown in Figure 6b reveal a higher weight loss with the higher content of fly ash in the initial formulation. The surfactant influence is not taken into account as it is added in an aqueous solution 1/10, so the total weight loss attributed to it could not exceed 0.4%. 

It is quite challenging to identify the weight losses in the hardened foams, as they are the result of decomposition reaction of several compounds. Two principal changes, however, could be reasonably explained. The gradual weight loss up to 500 °C was attributed mainly to the evaporation of physical bonded and combined water in the gel, whereas the loss at 500–700 °C is thought to correspond mainly to the carbon combustion and secondly to the final dehydration of the C–S–H and N–A–S–H compounds [38]. Beyond 700 °C, the weight losses were stabilized in all the samples, and only the sulphate decomposition from the initial fly ash could be noticed [37].

**Figure 7:**
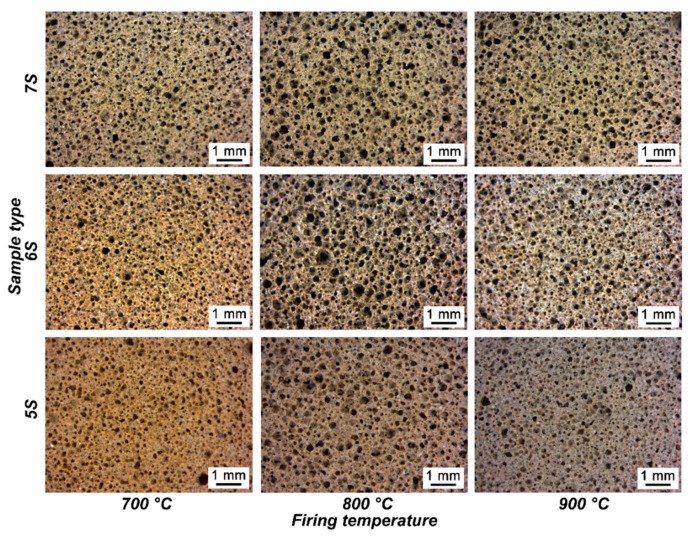
Morphological characterization of the fired foams with different SiO_2_/Al_2_O_3_ molar ratio, after firing.

After the heat treatment, all the samples resulted in well-sintered bodies, illustrated in Figure 7. Homogeneous well distributed round pores, with diameters from 10–30 μm, remained from the initial structure (after hardening). Even if the treatment temperature between 700–900 °C was well beyond the softening point of glass (around 650 °C), no coalescence of the cells, by viscous flow, could be observed, as an effect of the presence of crystal inclusions evolved from FA as well as from glass/FA interactions, and that increased the apparent viscosity on the melt. 

**Figure 8:**
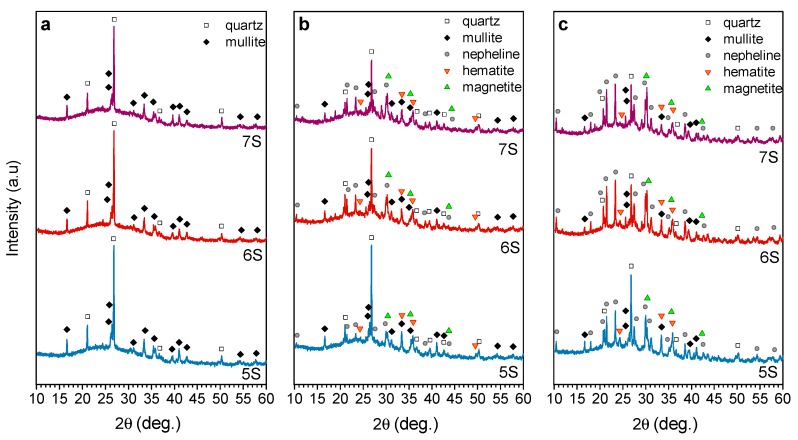
X-ray diffraction patterns of the fired foams with different compositions; (**a**) 700 °C; (**b**) 800 °C; and (**c**) 900 °C.

The evolution of the crystalline phases upon firing is illustrated by the X-ray diffraction patterns in Figure 8. The samples treated at 700 °C (Figure 8a) show that quartz (SiO_2_; PDF#083-0539) and mullite (Al_4_SiO_8_; PDF#079-1275) remained as the only identifiable phases. At this temperature, just above the softening point, SLG could just ‘glue’ FA particles, with limited chemical interaction. By contrast, softened SLG dissolved some quartz, especially for low FA/glass ratio (sample 7S), starting from 800 °C (Figure 8b). At 800 °C and, above all, at 900 °C, the SLG/FA interaction was substantial enough to lead to the precipitation of nepheline (Na_6.65_Al_6.24_Si_9.76_O_32_, PDF#083-2372), a quite typical crystalline phase formed upon transformation of geopolymers at high temperature [39,40,41].

The increase of temperature also determines the precipitation of iron oxides, in the form of hematite (Fe_2_O_3_, PDF#89-0691) and magnetite (Fe_3_O_4_, PDF#89-0691). Its relative abundance is difficult to quantify. However, a significant increase of the magnetite can be noticed after the heat treatment at 900 °C.

**Figure 9:**
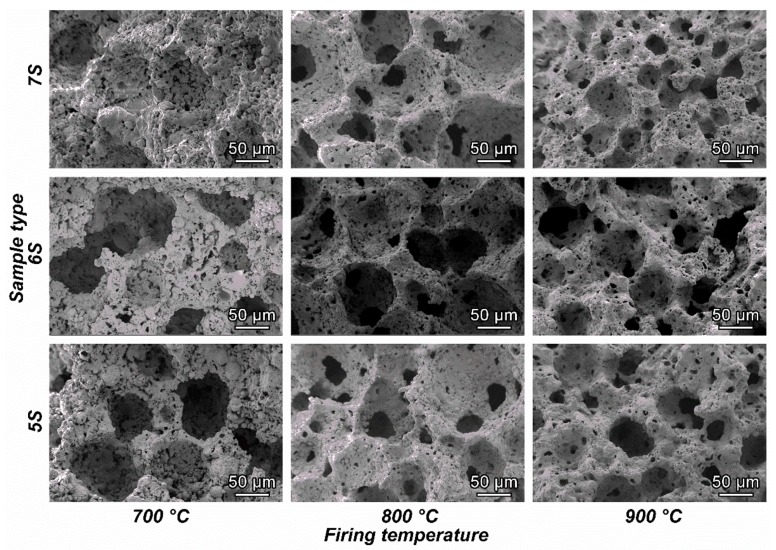
Microstructural details of glass–ceramic foams with different SiO_2_/Al2O_3_ molar ratio, after firing.

Density and porosity data of fired samples are shown in Table 2. High total porosity values are achieved, and it may be noted that the porosity remained mostly open for samples of the 5S and 6S series. Samples with higher glass content (7S) exhibited a more substantial reduction of both overall porosity and open porosity by increasing firing temperature, as an effect of viscous flow. The viscous flow is evident from the microstructural details in Figure 9: Especially samples with relatively low glass content (5S and 6S) were evidently poorly sintered at 700 °C; passing to 800 and 900 °C effectively led to the mixing of the constituents and to the formation of a uniform solid phase.

**Figure 10:**
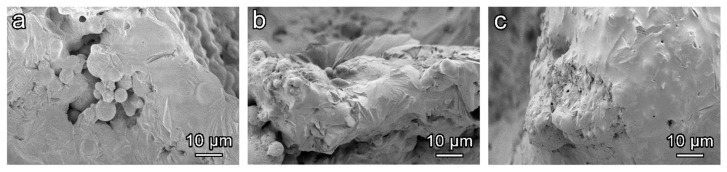
High magnification details of the foams struts after firing at 800 °C; (**a**) 5S, (**b**) 6S, and (**c**) 7S.

The densification with increasing glass content is even more evident considering the morphology of cell struts, shown in Figure 10, for samples fired at 800 °C. It can be appreciated that in the sample with lower glass amount, 5S (Figure 10a), some spherical particles, recognized as fly ash, remained visible; by contrast, they were completely incorporated with higher glass content, with evidence of formation of fibrous crystals. 

The strength data reported in Table 2 are interesting, above all, for the correlation with density data. In fact, any serious comparison should take into account the downscaling of mechanical properties operated by porosity. For samples possessing an abundant overall porosity, with very limited closed porosity (samples of the 5S and 6S series, 7S only fired at 900 °C), we may consider a simplified expression of the model proposed by Gibson and Ahsby [42], for the crushing strength (σ_c_) of bending-dominated cellular structures, as follows:

σ_c_ ≈ σ_bend_·C·(ρ_rel_)^3/2^ (1)

where the relative density (ρ_rel_) is defined as ρ_rel_ = 1−P/100 (P is the total porosity), C is a dimensionless constant (~0.2), and σ_bend_ is the bending strength of the solid phase. If we consider the relative densities of samples 5S and 6S fired at 800–900 °C, and of sample 7S fired at 700 °C, ranging from 0.3 to 0.35, the observed crushing strength values (marked in bold character in Table 2) are remarkable. In fact, these values correspond to a bending strength of the solid phase well above 100 MPa, in turn exceeding the bending strength of most glasses [43].

Table 3 shows the chemical analysis of the leachate of the glass–ceramic foams after the heat treatment at 800 and 900 °C and the initial raw materials. According to EN-12457, the leachates of the selected samples are well below the thresholds for inert materials in all the analyzed metal ions, with just one exception. The 5S sample treated at 800 °C exhibited a release of molybdenum ions slightly above the limit, but it should be considered as safe in any case, observing that the leaching tests were applied on highly porous samples, featuring a high surface-to-volume ratio, i.e., in particularly severe conditions. 

The stabilization of the potential pollutants present in the initial waste materials supports the possible use of the waste-derived glass–ceramic foams as environmentally friendly materials for thermal and acoustic insulation. For this reason, selected samples were produced on a semi-industrial scale, given that the proposed method with alkali activation and foaming is beneficial in the manufacturing of large panels. In addition, low-temperature foaming does not imply any geometrical limitation. The firing temperature (800 °C) is far below that adopted in the case of cheapest ceramics for building applications, such as clay bricks.

**Figure 11:**
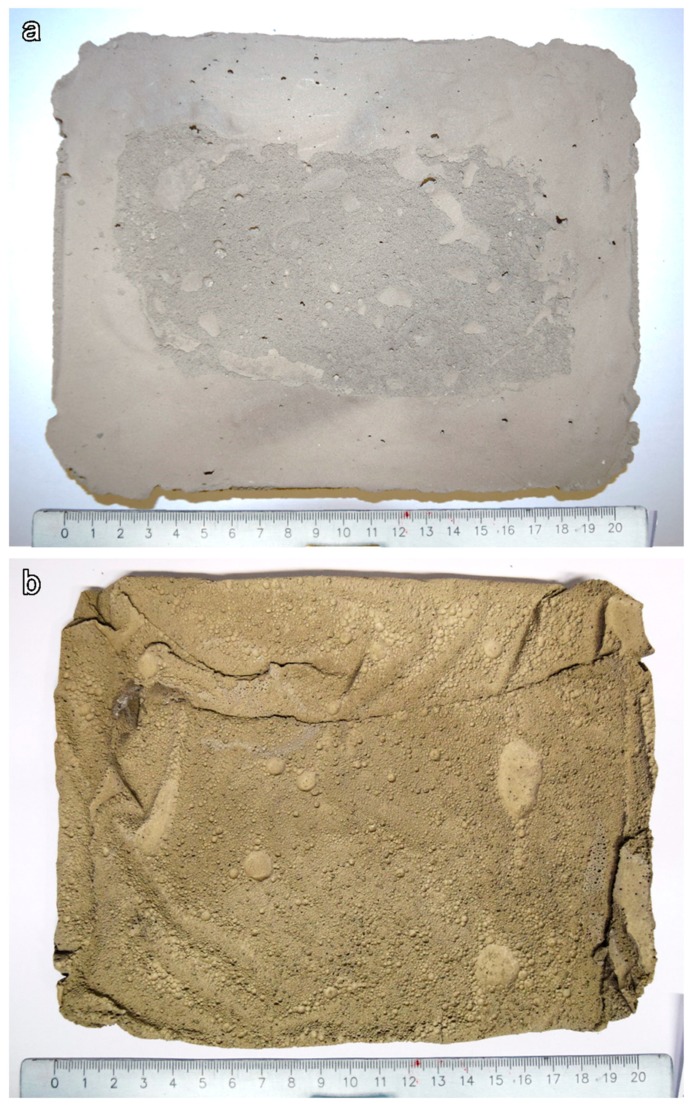
General view of lightweight panels; (**a**) 6S hardened panel; (**b**) heat treated panel at 800 °C.

The overall aspect of the 5S hardened lightweight panels produced with a big mold, after 24 h of post-foaming, is illustrated in Figure 11a (thickness of about 20 mm). The panels show good consistency with no cracks and acceptable mechanical properties, which make them easy to handle. After firing at 800 °C (Figure 11b), the overall structure remained unaltered, as observed on laboratory scale (the sample shown here was not cut or rectified). The faster heat treatment applied in a semi-industrial furnace did not cause the formation of any visible cracks (no preheating was applied to the samples, since they were inserted directly into the furnace). 

The thermal conductivity measured at 25 °C in the 5S samples treated at 800 °C was 0.163 ± 0.005 W·m^−1^·K^−1^. Such a relatively low value could be explained due to the low density and the particular cellular structure developed in the panels. Despite the open porosity, the reduced size and the homogeneous distribution probably had an advantageous effect, by reducing air convection [44]. These optimized foam panels could find applications in the building industry, as thermal insulators; moreover, the open-celled morphology presented by the ceramic foams along with the abundant content of iron oxide could support additional applications, e.g., as catalytic supports. 

## 4. Conclusions

We may conclude that:

- The technique ensures an excellent approach to produce glass–ceramic foams allowing the incorporation of high proportions of fly ash.
- This approach provides a recycling route to glass fraction currently landfilled, providing a solution to the landfill derived problems as well as a significant economic advantage.
- The possibility to use low alkali activator concentrations to produce a geopolymer-like gel, which acts as a binding phase, is demonstrated.
- The decomposition of the gel and the SLG/FA interactions upon firing promote the formation of the glass–ceramic foams.
- The developed glass–ceramic foams have high porosity, low thermal conductivity, and reasonable mechanical properties to be applied as thermal insulation materials.
- The chemical stability of the glass ceramic foams was assessed by leaching tests; the release of heavy metals remained below the threshold specification for inert materials.

## Figures and Tables

**Table 1:** Chemical composition (expressed in wt%) of the starting materials.

Oxide (wt%)	SiO_2_	Al_2_O_3_	Na_2_O	K_2_O	CaO	MgO	Fe_2_O_3_	TiO_2_
FA	54.36	24.84	0.83	3.03	2.56	2.06	8.28	1.07
SLG	70.5	3.2	12	1	10	2.3	0.42	0.07

**Table 2:** Density, porosity, and mechanical properties of the fired treated foams at different heating temperatures.

Sample	Firing Tª (°C)	Density, ρ (g/cm^3^)			Porosity (%)			σ_comp_ (MPa)
		Geometric	Apparent	True	Total	Open	Closed	
5	700	0.71 ± 0.01	2.28 ± 0.03	2.46 ± 0.01	71 ± 1	68 ± 2	2 ± 1	1.8 ± 0.2
	800	0.82 ± 0.01	2.32 ± 0.01	2.49 ± 0.01	67 ± 1	64 ± 1	2 ± 1	**5.1 ± 0.3**
	900	0.87 ± 0.01	2.36 ± 0.01	2.51 ± 0.01	65 ± 1	62 ± 1	2 ± 1	**7.4 ± 0.3**
6	700	0.54 ± 0.08	2.25 ± 0.02	2.42 ± 0.01	78 ± 2	75 ± 3	2 ± 1	1.9 ± 0.2
	800	0.66 ± 0.01	2.26 ± 0.02	2.43 ± 0.01	72 ± 1	70 ± 2	2 ± 1	**4.5 ± 0.2**
	900	0.71 ± 0.01	2.26 ± 0.01	2.45 ± 0.01	70 ± 3	68 ± 3	2 ± 1	**5.4 ± 0.4**
7	700	0.71 ± 0.01	2.31 ± 0.06	2.46 ± 0.01	71 ± 1	69 ± 2	2 ± 1	**3.8 ± 0.2**
	800	0.83 ± 0.01	2.13 ± 0.05	2.49 ± 0.01	66 ± 2	61 ± 2	6 ± 1	5.3 ± 0.4
	900	1.02 ± 0.3	2.08 ± 0.02	2.44 ± 0.01	58 ± 3	50 ± 4	7 ± 2	8.7 ± 0.6

**Table 3:** Leachate chemical analysis of selected samples and initial materials.

Element (ppm)	5S3M		6S3M		7S3M		Initial Materials		Limits [UE] (ppm)	
	800	900	800	900	800	900	FA	SLG	Inert material	Non-hazardous material
As	0.0316	0.0635	0.068	0.0503	0.0491	0.0795	<0.0049	<0.0049	0.5	2
Ba	>al	>al	>al	>al	0.0672	0.1108	<0.000	>al	20	100
Cd	<0.0002	<0.0002	<0.0002	<0.0002	<0.0002	<0.0002	<0.0002	0.001	0.04	1
Cr	0.3406	0.0805	0.0255	0.0598	0.0689	0.3001	0.4672	0.0043	0.5	10
Cu	0.0029	0.0183	0.0024	0.0065	0.0053	0.0207	0.0282	0.0036	2	50
Hg	0.0032	<0.0004	0.0006	<0.0004	0.0020	0.0017	0.8983	<0.0004	0.01	0.2
Mo	0.5324	0.0472	0.2107	0.0087	0.1973	0.2435	<0.0004	0.007	0.5	10
Ni	<0.0014	<0.0014	<0.0014	<0.0014	<0.0014	<0.0014	<0.0014	<0.0014	0.4	10
Pb	<0.0047	<0.0047	<0.0047	<0.0047	<0.0047	<0.0047	<0.0047	0.018	0.5	10
Se	0.0133	<0.0122	0.0255	<0.0122	0.0226	<0.0122	<0.0122	0.018	0.1	0.5
Zn	<0.0203	<0.0203	<0.0203	<0.0203	<0.0203	<0.0203	<0.0203	0.088	4	50
pH	9.4	8.6	8.2	7.7	8.0	7.6				
Abbreviation: >al, above detection limit										
